# Transcriptome differential expression analysis of defoliation in different lemon varieties under drought treatment

**DOI:** 10.1371/journal.pone.0299261

**Published:** 2024-04-18

**Authors:** Meichao Dong, Tuo Yin, Dongguo Zhou, Hanyao Zhang, Fan Yang, Shaohua Wang, Chunrui Long, Xiaomeng Fu, Hongming Liu, Lina Guo, Junyan Gao

**Affiliations:** 1 Institute of Tropical and Subtropical Cash Crops, Yunnan Academy of Agricultural Sciences, Baoshan, China; 2 The Key Laboratory of Biodiversity Conservation of Southwest China, National Forestry and Grassland Administration, College of Forestry, Southwest Forestry University, Kunming, China; South China Agricultural University, CHINA

## Abstract

’Allen Eureka’ is a bud variety of Eureka lemon with excellent fruiting traits, but severe winter defoliation affects the following year’s yield, and the response mechanism of lemon defoliation is currently unknown. Two lemon cultivars (’Allen Eureka’ and ’Yunning No. 1’) with different defoliation traits were used as materials to investigate the molecular regulatory mechanisms of different leaf abscission periods in lemons. The petiole abscission zone was collected at three different defoliation stages, namely, the predefoliation stage (k15), the middefoliation stage (k30), and the postdefoliation stage (k45). Transcriptome sequencing was performed to analyze the gene expression differences between these two cultivars. A total of 1141, 2695, and 1433 differentially expressed genes (DEGs) were obtained in k15, k30, and k45, respectively, and the number of DEGs in k30 was the largest. GO analysis revealed that the DEGs between the two cultivars were mainly enriched in processes related to hydrolase activity, chitinase activity, oxidoreductase activity, and transcription regulator activity in the defoliation stages. KEGG analysis showed that the DEGs were concentrated in k30, which involved plant hormone signal transduction, phenylpropanoid biosynthesis, and biosynthesis of amino acids. The expression trends of some DEGs suggested their roles in regulating defoliation in Lemon. Seven genes were obtained by WGCNA, including sorbitol dehydrogenase (*CL9G068822012_alt*, *CL9G068820012_alt*, *CL9G068818012_alt*), abscisic acid 8’-hydroxylase (*CL8G064053012_alt*, *CL8G064054012_alt*), and asparagine synthetase (*CL8G065162012_alt*, *CL8G065151012_alt*), suggesting that these genes may be involved in the regulation of lemon leaf abscission.

## 1. Introduction

Lemon (*Citrus limon* (L.) Burm. F.) is an evergreen fruit tree of the citrus genus (*Citrus medica* L.) in the family Rutaceae. It may be a natural hybrid of lime (C. *aurantium*) and citron (C. *medica*) [1, 2], originating in northeastern India, northern Burma, and southern China [3]. According to FAO data for lemons and limes in 2021, the global harvested area was 1338321 hectares with a total production of 20828739 tons, with the top five producers currently being India, Mexico, China, Argentina, and Brazil. The global lemon industry includes India, Mexico, China, and other major producing countries "racing together bridle to bridle". The scale of lemon production is a continuous growth momentum. Lemon is an evergreen nondeciduous fruit tree, and the production of ’Allen Eureka’ lemon was found to be high yield, but under drought stress, defoliation is significantly higher than that of other varieties, resulting in many loss of organic nutrients, delayed flowering of the tree in the following year, and a decline in yield. If the causes of ’Allen Eureka’ lemon defoliation can be clarified, the methods to reduce the rate of ’Allen Eureka’ lemon defoliation and maintain the yield can be further produced, which is of great significance for the adjustment of lemon varieties and the economic development of active lemon growing production areas.

Lemon defoliation is a manifestation of damage in plants subjected to abiotic stresses such as drought and low temperatures and a defense mechanism to reduce water loss and protect the tree; however, its intrinsic mechanism remains unclear. A new perspective on drought-induced organ abscission was provided using yellow lupin as a study subject. Organ abscission under drought stress is a cellular remodeling behavior due to cell wall modification, and the process mainly relies on the reorganization of methylated high galacturonic acid (HG) in the isolated layer and the upregulation of pectin methyl esterase (PME) and polygalacturonase (PG) [4]. Studies have shown that the defoliation caused by rewatering after drought in citrus is mainly because, under drought conditions, the root system can accumulate a large amount of 1-aminocyclopropane-1-carboxylic acid (ACC), which serves as a precursor for the synthesis of ethylene. After rewatering, ACC is transferred from the root system to the shoots for ethylene synthesis, resulting in citrus defoliation [5]. In addition, scientists have found that water stress can cause citrus leaves to fall off because their roots accumulate high levels of abscisic acid [6].

Transcriptome sequencing is high-throughput sequencing of mRNA from a species that dynamically reflects the level of gene transcription, identifies and quantifies both rare and regular transcripts, and provides sample-specific information on the sequence structure of transcripts [7]. Currently, transcriptome sequencing is widely used in plant organ abscission studies. In the early stage of melon fruit ripening, petiole isolation is downregulated by MAD-box, AP2/ERF, and Aux/IAA transcription factors and upregulated by transcription factors such as homeobox, zinc finger bZIP, and WRKY. However, in the later regulation stage, isolation is upregulated by MYB transcription factors, so melon isolation is differentially genetically regulated in the early and late stages of ripening [8]. Gao et al. [9] performed gene expression profiling and screening to identify 2571 transcripts associated with abscission and the gene RhIAA16 in response to abscission at the petal abscission site during three periods of moonflower, with the highest expression at the abscission initiation stage where abscission occurs, and the expression was downregulated after the initiation of abscission, which is sufficient to indicate that the RhIAA16 gene is associated with the initiation of abscission. Evergreen citrus was treated with ethylene glycol, and transcriptome sequencing was performed on the treated delaminated sites. Analysis of the RNA-Seq results showed that cell wall-modifying enzyme-encoding genes, stress-related genes, pathogen-related genes, MAPK kinase-related genes, and transcription factors, as well as ethylene biosynthesis and signaling genes, were significantly expressed in the delaminated sites [10, 11].

In this study, two lemon varieties were selected: ’Allen Eureka’, which has obvious defoliation under drought stress, and ’Yunning No. 1’, which does not have obvious defoliation under drought stress. Physiological and transcriptomic studies of the two lemon varieties during different periods of drought stress were carried out to provide reliable data for the screening of lemon defoliation candidate genes and the analysis of defoliation pathways, as well as providing a theoretical basis and scientific evidence for the selection and breeding of high-quality and highly resistant lemon varieties.

## 2.Materials and methods

### 2.1. Experimental materials

The test materials were selected as scions of two lemon varieties, ’Allen Eureka’ and ’Yunning No. 1’, which were grafted onto the annual Poncirus trifoliate (L.) Raf rootstocks in November 2019. When the two lemon varieties reached an average plant height of 60 cm, the seedlings of the two lemon varieties with good growth conditions and similar sizes were selected for transplanting and cultivation. The cultivation container was a plastic bucket (the diameter of the mouth of the bucket was 60 cm, the diameter of the bottom of the bucket was 55 cm, and the height of the bucket was 60 cm). The cultivation substrate was coco coir: red loam: organic fertilizer = 6:3:1. For each variety, 180 plants were selected, and one was planted in each bucket. A total of 360 plants were grown and placed in the rain-sheltered arch shed of the Institute of Tropical Subtropical Cash Crops, Yunnan Academy of Agricultural Sciences, (97°87′E, 24°2′N), with a shed height of 3.2~3.5 m, during which regular management was carried out. Water control was conducted when the plant reached an average height of 200 cm and an average crown width of 150 cm or more.

### 2.2. Sampling

Drought stress was applied by the artificial water control method in bucket planting, and the soil relative humidity was measured using a Peng Yun remote soil thermohygrometer (model: S21A). The air relative humidity during the test period ranged from 70 to 89%, and the air temperature varied from 20°C to 32°C. The experiment was set up for three treatment periods of mild, moderate, and severe drought stress. Mild drought stress manifested as the older leaves began to fall off one after another, and the young leaves were slightly curled. At this time, the measured relative soil water content was 45±5% RH. Moderate drought stress manifested as the mature leaves began to fall off in large quantities, and the young leaves were curled in large quantities. At this time, the measured relative soil water content was 35±5% RH, and severe drought stress manifested as a reduction in the number of mature leaves that fell off, but a few plants appeared to be drying out of the young leaves. At this time, the measured relative soil water content was 28±5% RH.

Two lemon varieties, ’Allen Eureka’ and ’Yunning No. 1’, were selected with 150 plants each, and all plants were watered thoroughly on June 15, 2021. Then, the water supply was stopped. When the soil reached the relative water content range values of mild (labeled predefoliation stage k15), moderate (labeled middefoliation stage k30), and severe (labeled postdefoliation stage k45) drought stress, each variety was divided into nine groups (three groups for enzyme activity determination, three groups for hormone determination, and three groups for transcriptome determination). First, 1.0 g of middle and lower mature petioles of barrel-planted plants from different treatment periods were collected from the abscission zone (0.3–0.5 cm at the base of the petiole), and the samples were transferred to liquid nitrogen and quickly frozen after sample collection and placed at -80°C for freezing and preservation for future use.

### 2.3. Measurement of enzyme activities

A kit was used to determine superoxide dismutase (SOD) activity, peroxidase (POD) activity, catalase (CAT) activity, malondialdehyde (MDA) content, cellulase activity, and pectinase activity, which was repeated three times. The primary test instrument was an MD SpectraMax 190 full-wavelength enzyme labeler.

### 2.4. Hormone determination

In this experiment, the 1-aminocyclopropane-1-carboxylic acid (ACC), abscisic acid (ABA), auxin (IAA), and gibberellin (GA3) contents of two lemon varieties at three periods were determined using high-performance liquid chromatography-tandem mass spectrometry (HPLC‒MS/MS), which was repeated three times.

### 2.5. RNA extraction, cDNA library preparation, and transcriptome sequencing

The primary test instrument was an MD SpectraMax 190 full-wavelength enzyme labeler. Total RNA was extracted from 18 lemon samples that represent three biological replicates of two lemon cultivars at three defoliation stages (named Ak15-1, Ak15-2, Ak15-3, Ak30-1, AK30-2, Ak30-3, Ak45-1, Ak45-2, Ak45-3, Yk15-1, Yk15-2, Yk15-3, Yk30-1, Yk30-2, Yk30-3, Yk45-1, Yk45-2, and Yk45-3) using the RNAprep Pure Plant Kit (QIAGEN, Germany). The mRNA was enriched with magnetic beads with oligo (dT), breaking the mRNA into short fragments and using the mRNA fragments as templates for cDNA construction. The sequencing library was constructed and then sequenced with Illumina HiSeq to obtain the raw transcriptome sequencing data.

### 2.6. Transcriptome data analysis

The raw data were filtered using fastp software (https://github.com/OpenGene/fastp) to avoid a large number of junctions and low-quality data in the transcriptome data, and the filtering criteria were as follows: (1) removing reads containing junctions; (2) removing reads with an N-ratio of more than 10%; (3) removing reads with a low-quality ratio of bases higher than 50% (quality value less than 20); and finally using FastQC software for quality control of clean data. Finally, FastQC software was used to check the quality of the clean data, and the subsequent analysis was performed after passing the quality control.

The QC-qualified clean reads were compared to the reference genome C. limon L. Burm f. genome v1.0 (https://www.citrusgenomedb.org/Analysis/1470607) using HISAT2 v2.0.5 [12, 13], followed by StringTie [14] software. The transcript abundance degree of all genes was counted based on the clean data and the reference genome comparison. FPKM (fragments per kilobase of exon model per million mapped fragments) was calculated for each gene to quantify the expression of each gene. Differential gene analysis was performed using DESeq2 [15] in this study, and genes with |log2 (fold change)| ≥ 1 and p-value ≤ 0.05 were defined as significantly differentially expressed genes. In addition, we performed GO function and KEGG pathway enrichment analysis of differentially expressed genes using clusterProfiler (3.8.1) software.

### 2.7. RT‒qPCR analysis

The expression levels of six differentially expressed genes selected from the RNA-seq data were verified by RT‒qPCR using the cDNA samples used for RNA-Seq library construction. Primer 5.0 software was used to design primer pairs. The primer sequence was synthesized by Beijing Tian Yi Hui Yuan Biotechnology Co., Ltd., and the primer information is shown in Table 1. RT‒qPCR was performed using a Roche LightCycler 480 with the following procedure: 95°C for 5 min, followed by 40 cycles at 95°C for 10 s, 60°C for 10 s, and 60°C for 30 s. The housekeeping gene Actin was used as an internal control. The relative expression level was calculated by the 2^-△△Ct^ method.

**Table 1:** Differential gene primer sequences.

Gene ID	Forward primer(5′-3′)	Reverse primer(5′-3′)
*CL2G044126012_alt*	TGTGAGGAATGACCGTGGC	ACTTGTCGTGGAGCTTGTTGG
*CL7G062118012_alt*	TGCTCTGAACGATACGGTGC	CCTCTCACGTCAACACCTCCA
*CL2G044587012_alt*	ATTGGTACGAGGCAGAACGG	CGTCACGCTCAGAATGTTAGGA
*CL9G066930012_alt*	ACGGCAGCATTTGTGAAGGT	ATCACCCACGAGCATCCAGT
*CL3G046634012_alt*	CAACTCCGCCTAGCAAGACAC	CTCCCACAAGCATCCAATCTC
*CL8G065380012_alt*	CCAGAGCGATAGGCGACAAC	AGCAACACTTGTCCGTCGTCT
*Actin*	ACTCATCGTACTCAGCCTTTG	TGCACCCTGTTCTTCTTACTG

### 2.8. Statistics and analysis of experimental data

In this study, Excel 2016 was used for statistical processing of data as well as graphing; heatmaps of expression patterns were drawn using the Pheatmap package of R software; gene coexpression network analysis and visualization were performed using the WGCNAv1.69 package of R software.

## 3. Results

### 3.1. Changes in enzyme activities during different defoliation periods in the two lemon varieties

**Fig 1:**
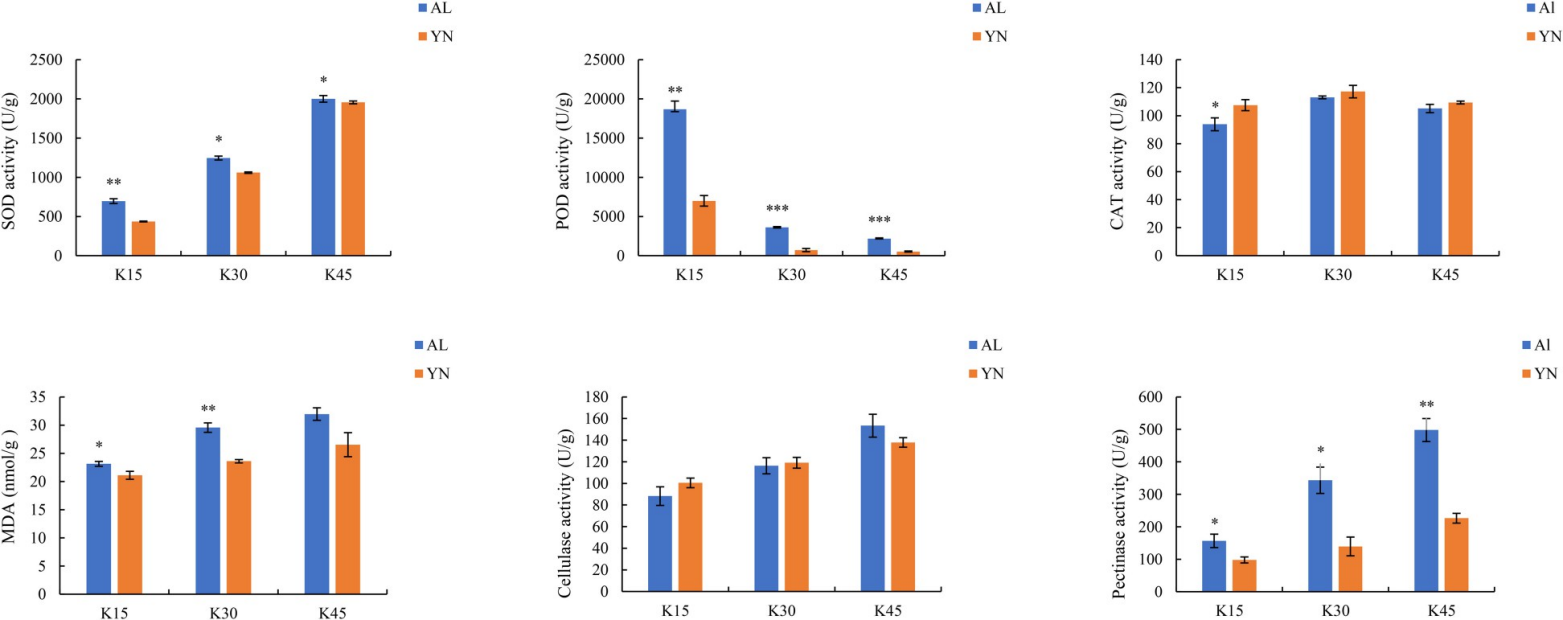
Changes in enzyme activities during different defoliation periods in two varieties, ’Allen Eureka’ and ’Yunning No. 1’ (Significance is the difference between the two varieties; * p < 0.05, ** p < 0.01, *** p < 0.001).

The SOD, MDA, cellulase, and pectinase activities of two lemon varieties, ’Allen Eureka’ and ’Yunning No. 1’, all gradually increased with the prolongation of drought stress. CAT activity increased and then decreased with the prolongation of drought stress, but the magnitude of change was not many in either variety. POD activity showed a significant decreasing trend with the prolongation of drought stress and was significantly lower in the mid-defoliation stage (k30) and postdefoliation stage (k45) than in the predefoliation stage (k15). In both varieties, the content of POD in ’Allen Eureka’ was highly significantly higher than that in ’Yunning No. 1’ at three different periods of defoliation, and the content of SOD, MDA, and pectinase was also significantly higher than that in ’YN’ and the differences between the two varieties were distinct (Fig 1).

### 3.2. Changes in endogenous hormone contents during different defoliation periods in the two lemon varieties

**Fig 2:**
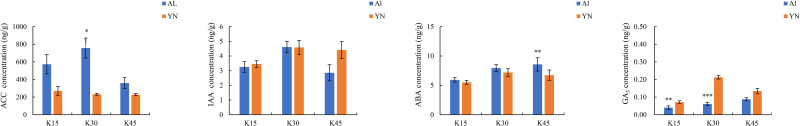
Changes in endogenous hormone contents during different defoliation periods in two lemon varieties, ’Allen Eureka’ and ’Yunning No. 1’ (Significance is the difference between the two varieties; * p < 0.05, ** p < 0.01, *** p < 0.001).

As shown in Fig 2, the ACC content of ’Allen Eureka’ showed a trend of increasing and then decreasing with the prolongation of drought stress. However, the ACC content of ’Yunning No. 1’ remained unchanged during the three defoliation periods. The contents of IAA in both ’Allen Eureka’ and ’Yunning No. 1’ showed a trend of increasing and then decreasing with the prolongation of drought stress. The ABA content of ’Allen Eureka’ showed a gradual increase with drought stress duration, while the ABA content of ’Yunning No. 1’ showed an increase followed by a slight decrease with drought stress duration. The GA3 content of ’Allen Eureka’ showed a gradual increase with the prolongation of drought stress, the GA3 content of ’Yunning No. 1’ showed an increase and then a decrease with the prolongation of drought stress, and the difference between the two varieties was significant.

### 3.3. Statistics of off-region transcriptome sequencing data

**Fig 3:**
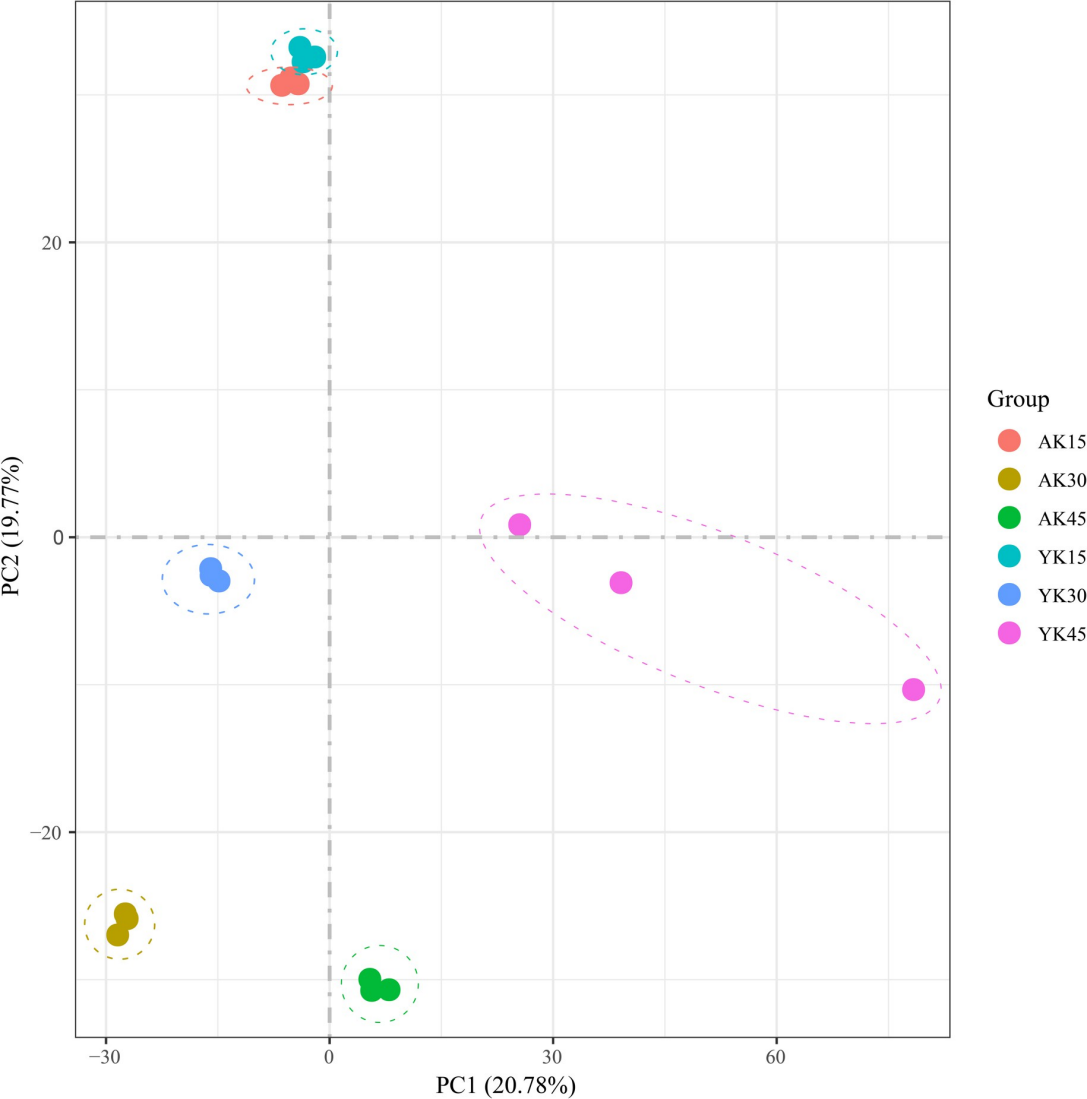
Principal component analysis (PCA) of differentially expressed genes in ’Allen Eureka’ and ’Yunning No. 1’ lemons at each defoliation period.

After sequencing quality analysis, 18 libraries were constructed from three replicates in three periods of ’Allen Eureka’ and ’Yunning No. 1’. A total of 115.73 Gb of clean data was obtained for 18 samples with Q30 > 89.63%. The number of raw reads ranged from 37773510 to 46471732. Both clean and uniquely mapped reads were matched by more than 70% (S1 Table), indicating that the reference genome was selected appropriately. The Pearson correlation coefficients between biological replicates for each sample ranged from 0.62 to 0.97. Eighteen samples were subjected to principal component analysis. After dimensionality reduction, it was found that the samples of the same variety and the same period were clustered together (Fig 3), and the results of the analysis showed that the reproducibility of all the samples used for this experiment was good.

### 3.4. Number of differentially expressed genes at different defoliation stages in the two lemon varieties

**Fig 4:**
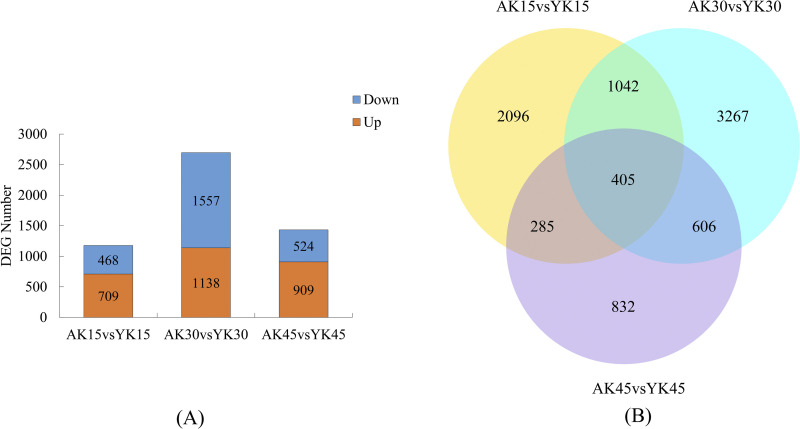
**(A)** Number of DEGs between ’AL’ and ’YN’ for each defoliation period. DEGs were screened according to |log2 (Fold change)| ≥ 1 and padj ≤ 0.05. **(B)** Venn diagram of ’Allen Eureka’ and ’Yunning No. 1’ for each defoliation period.

Two-by-two comparisons of ’Allen Eureka’ and ’Yunning No. 1’ genotypes were performed at the predefoliation, middefoliation, and postdefoliation stages of the two lemon varieties to identify lemon defoliation-related genes. In ’Allen Eureka’ relative to ’Yunning No. 1’, 1177 genes were significantly differentially expressed, 709 genes were significantly upregulated, and 468 genes were significantly downregulated in the predefoliation stage (k15). At the middefoliation stage (k30), 2695 genes were significantly differentially expressed, 1138 genes were significantly upregulated, and 1557 genes were significantly downregulated. At the postdefoliation stage (k45), 1433 genes were significantly differentially expressed, with 909 genes significantly upregulated and 524 genes significantly downregulated (Fig 4A), with the most differentially expressed genes in the middefoliation stage (k30) out of the three periods (S2 Table). A Venn diagram (Fig 4B) was plotted based on all the differentially expressed genes in the three periods, and a total of 405 differentially expressed genes in the three periods were compared with 2096 (k15), 3267 (k30), and 832 (k45) genes expressed in only one period.

### 3.5. GO enrichment analysis of differentially expressed genes at different defoliation stages in two lemon varieties

**Fig 5:**
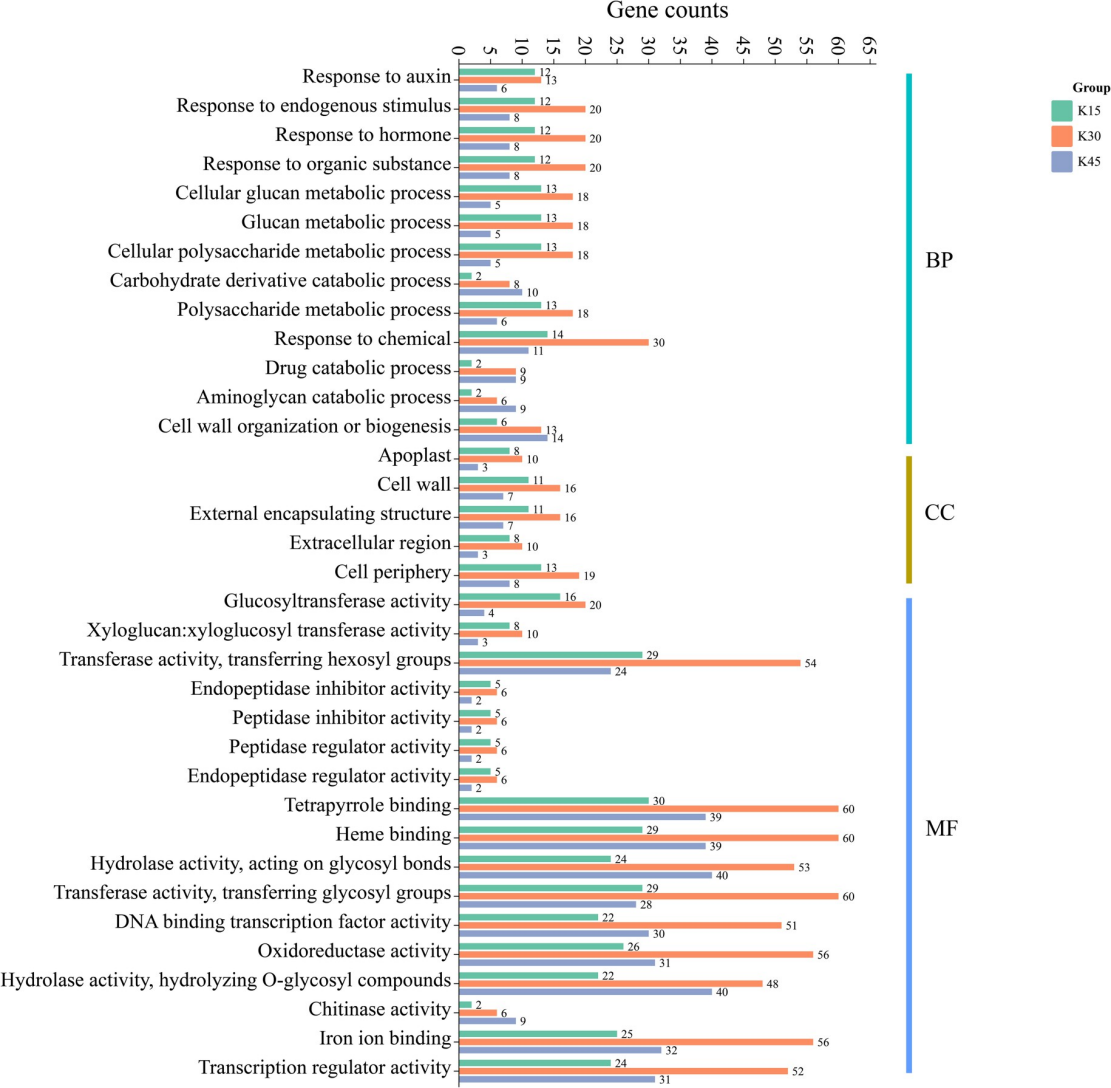
GO functional enrichment of differentially expressed genes in ’Allen Eureka’ and ’Yunning No.1’ lemons at each defoliation period.

Furthermore, GO enrichment analysis showed that 186 (predefoliation stage k15), 405 (middefoliation stage k30), and 124 (postdefoliation stage k45) DEGs were annotated to the GO database for ’Allen Eureka’ and ’Yunning No. 1’ lemons in the three periods, respectively. According to the significance level of GO term enrichment, the differentially expressed genes of the two varieties were mainly enriched in the molecular functional classifications of hydrolase activity, chitinase activity, glucosyltransferase activity, xyloglucosyl transferase activity, transferring hexosyl groups, oxidoreductase activity, iron ion binding, and transcription regulator activity, classification of cellular components of the apoplast, cell wall, external encapsulating structure, extracellular region, and cell periphery. In addition, the biological processes were categorized as aminoglycan catabolic process, carbohydrate derivative catabolic process, response to auxin, response to endogenous stimulus, response to hormone, response to organic substance, etc. (Fig 5). The related differential genes in molecular functions, cellular components, and biological process classifications were significantly different in all three periods, with most of them enriched to significantly higher differential genes in the Mid-defoliation stage (k30) GO than in Predefoliation stage (k15) and Postdefoliation stage (k45).

### 3.6. Metabolic pathway analysis of differentially expressed genes at different defoliation stages in two lemon varieties

#### 3.6.1 KEGG enrichment analysis

**Fig 6:**
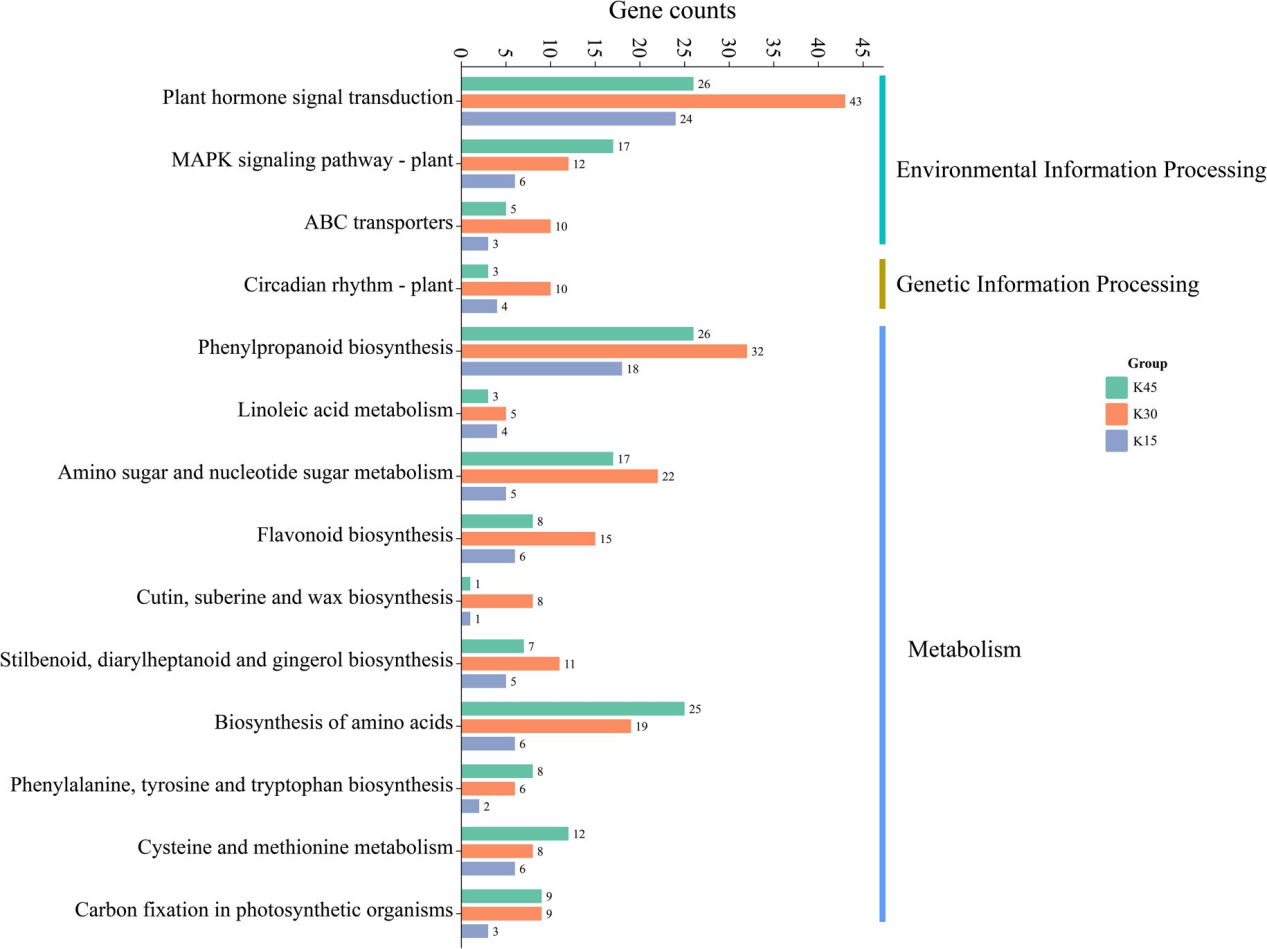
Enrichment of KEGG metabolic pathways of differentially expressed genes in ’Allen Eureka’ and ’Yunning No.1’ lemons at each defoliation period.

The results of KEGG metabolic pathway enrichment of differentially expressed genes between two lemon varieties, ’Allen Eureka’ and ’Yunning No. 1’, at three periods (Fig 6) showed that the number of differentially expressed genes and related metabolic pathways enriched at the mid-defoliation stage (k30) was the highest, mainly focusing on plant hormone signal transduction, phenylpropanoid biosynthesis, amino sugar and nucleotide sugar metabolism, biosynthesis of amino acids, flavonoid biosynthesis, and so on. The number of differential genes and related metabolic pathways enriched in the postdefoliation stage (k45) was high, focusing on plant hormone signal transduction, phenylpropanoid biosynthesis, biosynthesis of amino acids, amino sugar, and nucleotide sugar metabolism, MAPK signaling pathway-plant, cysteine and methionine metabolism pathways. Few metabolic pathways were enriched in the predefoliation stage (k15), mainly focusing on plant hormone signal transduction, phenylpropanoid biosynthesis, and linoleic acid metabolism pathways.

#### 3.6.2 Differentially expressed genes of the plant hormone signal transduction pathway

**Fig 7:**
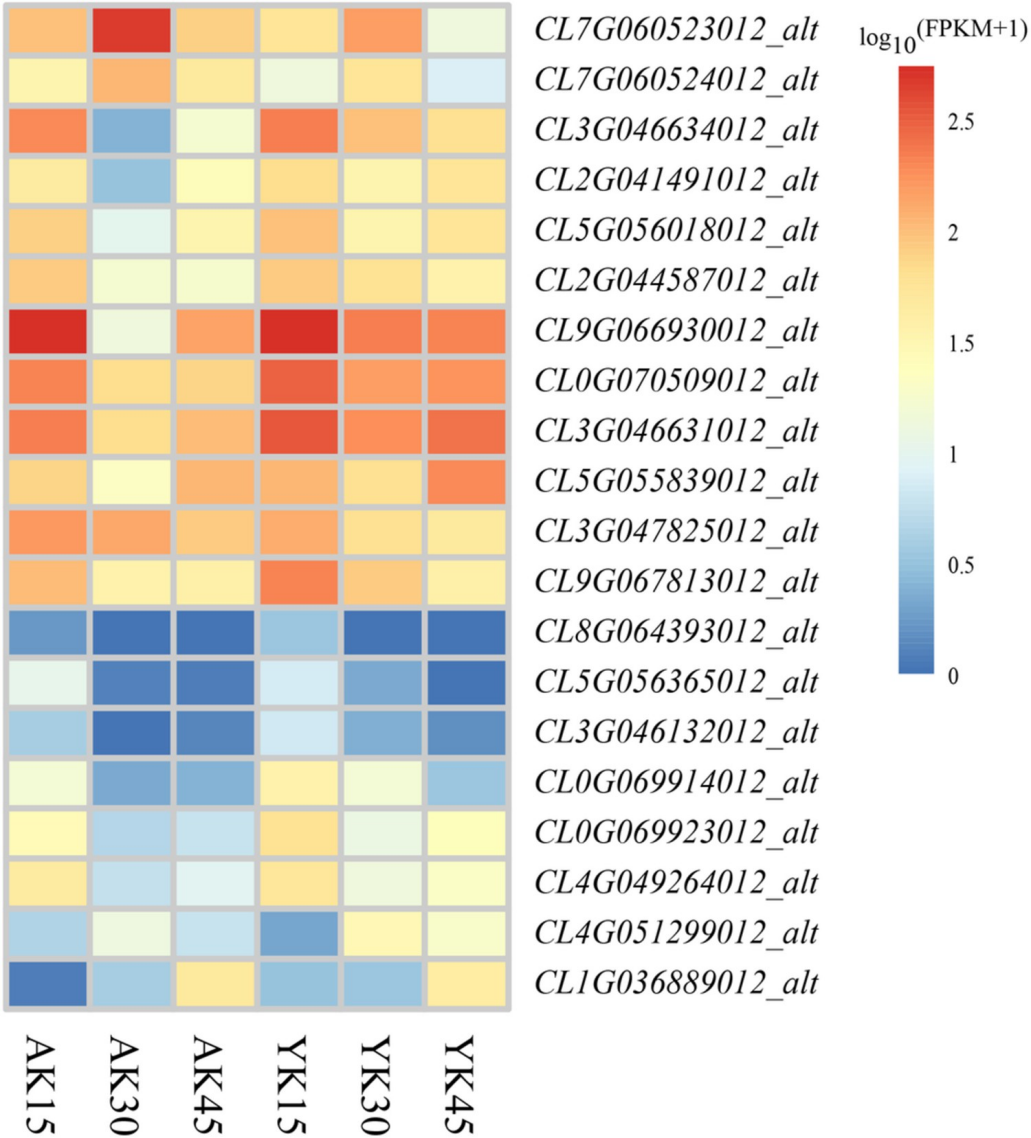
Expression characteristics of differentially expressed genes of the plant hormone signal transduction pathway in ’Allen Eureka’ and ’Yunning No. **1’ lemons at each defoliation period.** (The horizontal coordinates of the graphs in the heatmap are the sample names, and the vertical coordinates are the values of the differentially expressed genes after FPKM normalization; the redder the color is, the higher the expression, and the bluer the color is, the lower the expression).

The KEGG enrichment analysis revealed that the differentially expressed genes between ’Allen Eureka’ and ’Yunning No. 1’ lemons were more abundant in all three periods of the plant hormone signal transduction pathway, and comparing the three periods, the mid-defoliation stage (k30) had the most differentially expressed genes in the plant hormone signal transduction pathway. Among them, the relative expression of auxin transporter proteins (*CL7G060523012_alt* and *CL7G060524012_alt*) was significantly elevated in the mid-defoliation stage (k30) compared with that in the predefoliation stage (k15) in both cases, and the expression of ’Allen Eureka’ was significantly higher than that of ’Yunning No. 1’. The auxin response factors AUX/IAA (*CL5G056018012_alt* and *CL9G066930012_alt*), SAUR (*CL0G069923012_alt*), GH3 (*CL2G041491012_alt* and *CL5G056365012_alt*) and the GRAS family protein DELLA protein GAI (*CL4G049264012_alt*) as well as the two-component response regulator (*CL9G067813012_alt*) were all significantly lower in relative expression at the mid-defoliation stage (k30) than at the predefoliation stage (k15). The expression level of ’Allen Eureka’ was significantly lower than that of ’Yunning No. 1’ (Fig 7).

### 3.6.3 Differentially expressed metabolic pathway genes

**Fig 8:**
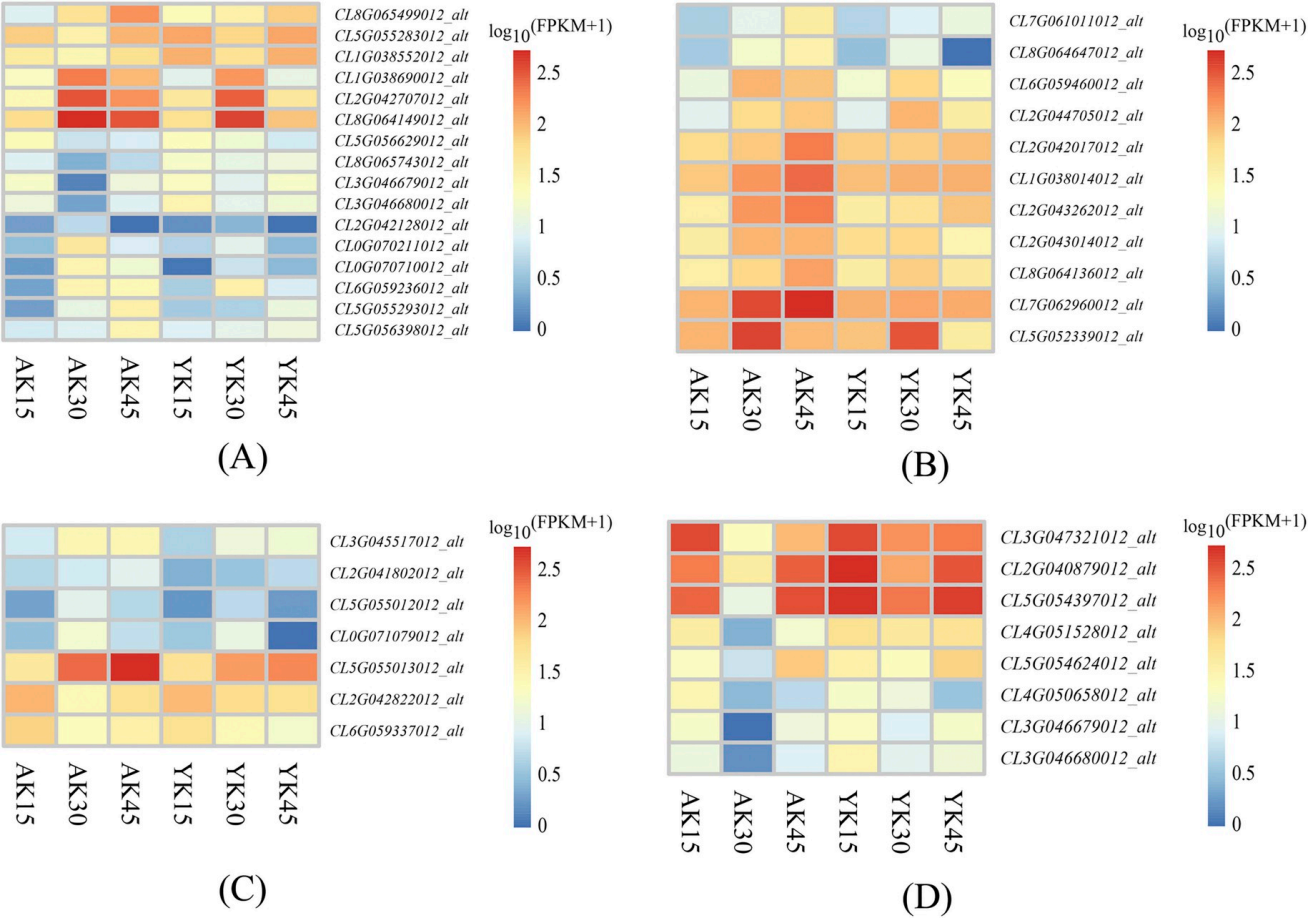
Expression characteristics of differentially expressed genes of metabolic pathways in each defoliation period of the ’Allen Eureka’ and ’Yunning No. 1’ lemons. (A) Phenylpropanoid biosynthesis; (B) Biosynthesis of amino acids; (C) Amino sugar and nucleotide sugar metabolism; (D) Flavonoid biosynthesis. (The horizontal coordinates of the graph in the heatmap are the sample names, and the vertical coordinates are the values of the differentially expressed genes after normalization of FPKM; the redder the color, the higher the expression, and the bluer the expression, the lower the expression).

As a result of KEGG enrichment analysis, the differentially expressed genes between the ’Allen Eureka’ and ’Yunning No. 1’ lemons were also relatively concentrated in metabolic pathways, especially phenylpropane biosynthesis (Fig 8A), amino acid biosynthesis (Fig 8B), aminoosugar and nucleotide sugar metabolism (Fig 8C), and flavonoid biosynthesis (Fig 8D). The expression of peroxidase (*CL5G056629012_alt*, *CL8G065499012_alt*, *CL0G070710012_alt*), β-glucosidase (*CL5G056398012_alt*, *CL2G042128012_alt*), and transferase (*CL5G055293012_alt*, *CL0G070211012_alt*) in the phenylpropane biosynthesis pathway differed considerably among the three defoliation periods. Among them, β-glucosidase (*CL2G042128012_alt*) was significantly increased in the mid-defoliation stage, and (*CL5G056398012_alt*) was significantly increased in the postdefoliation stage. The expression of ’Allen Eureka’ was significantly higher than that of ’Yunning No. 1’ in both periods. The relative expression of *CL5G056629012_alt* in peroxidase was highest in the predefoliation stage, and it was significantly decreased in the mid-defoliation stage and postdefoliation stage, whereas *CL8G065499012_alt* and *CL0G070710012_alt* had the lowest relative expression in the predefoliation stage and gradually increased in the mid-defoliation stage and postdefoliation stage. The expression of ’Allen Eureka’ was significantly higher than that of ’Yunning No. 1’ in both the mid- and postdefoliation stages. Glycosyltransferase *CL5G052339012_alt* in the biosynthetic pathway of amino acids showed a significant increase in expression at the mid-defoliation stage, and all declined at the postdefoliation stage. The relative expression of glyceraldehyde 3-phosphate dehydrogenase *CL7G062960012_alt* was significantly higher in the mid- and post-defoliation stages of ’Allen Eureka’ than in ’Yunning No. 1’. Both chitinase (*CL5G055013012_alt*, *CL5G055012012_alt*) and galactosidase (*CL3G045517012_alt*) in amino sugar and nucleotide sugar metabolism were most abundantly expressed in the mid-defoliation stage, and they were significantly higher in ’Allen Eureka’ than in ’Yunning No. 1’. Cytochrome P450 (*CL5G054397012_alt* and *CL5G054624012_alt)* in the flavonoid biosynthesis pathway was least expressed in the mid-defoliation stage and was significantly lower in ’Allen Eureka’ than in ’Yunning No. 1’.

### 3.7. Construction of gene coexpression networks

**Fig 9:**
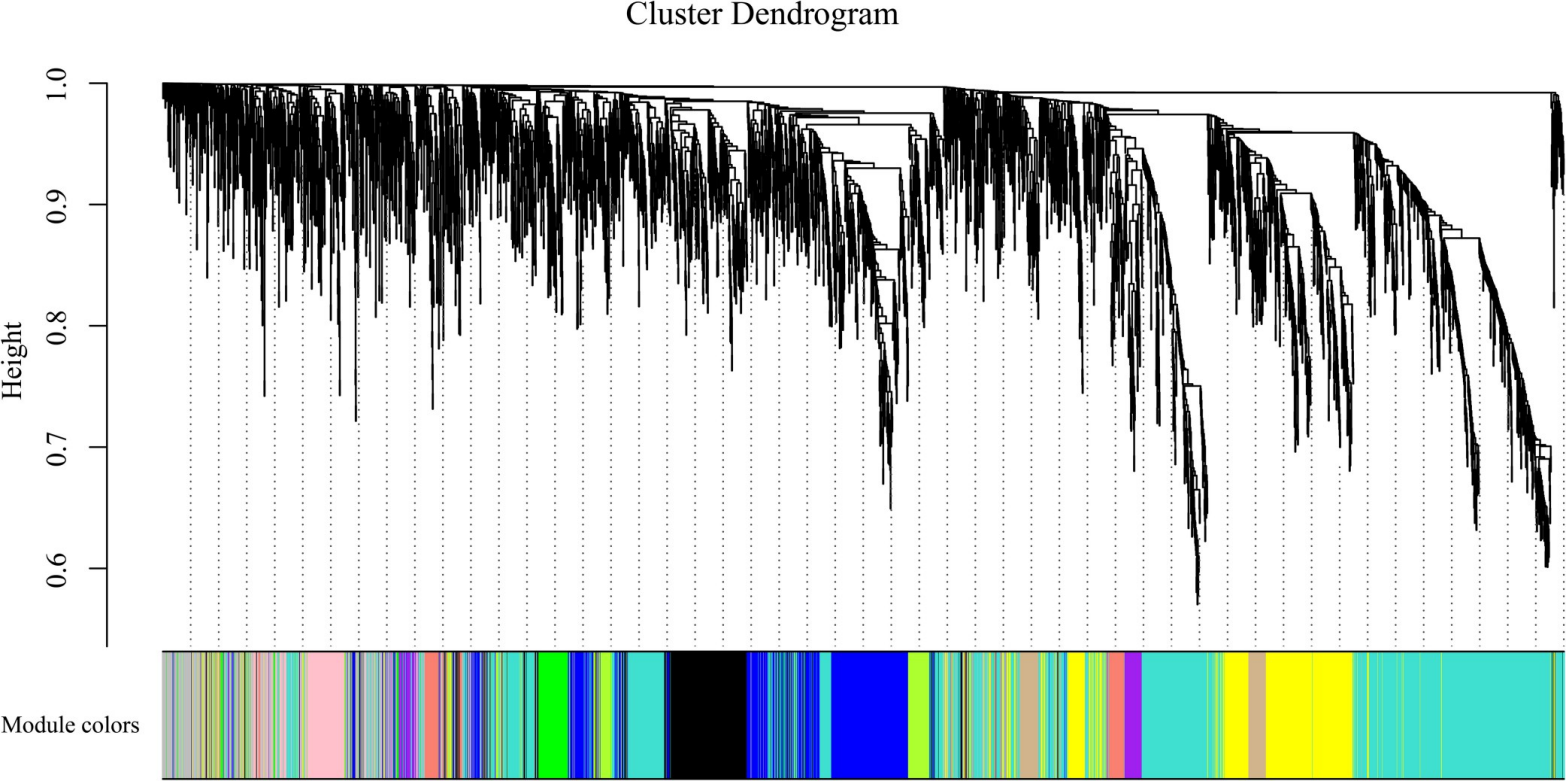
Gene clustering tree and module cuts (merging gene modules with similar expression patterns; Different colors represent different modules, the number of genes assigned in a module is clustered according to their expression in a correlation, and genes with higher clusters are assigned to a module).

To gain insight into the coexpressed genes of the two lemon varieties at three defoliation stages (k15, k30, and k45) and to mine genes related to defoliation height, WGCNA was performed. By screening the weight values, β = 15 was finally chosen to construct the network. The dynamic shear tree method was used to merge the modules with a similar expression. Sixteen coexpression modules were obtained (Fig 9), where the turquoise module contained the highest number of genes, 1551 genes, followed by the blue module containing 1121 genes, and the lowest number of genes within the midnight blue module containing 43 genes, and the average number of genes contained in each module was 506.

**Fig 10:**
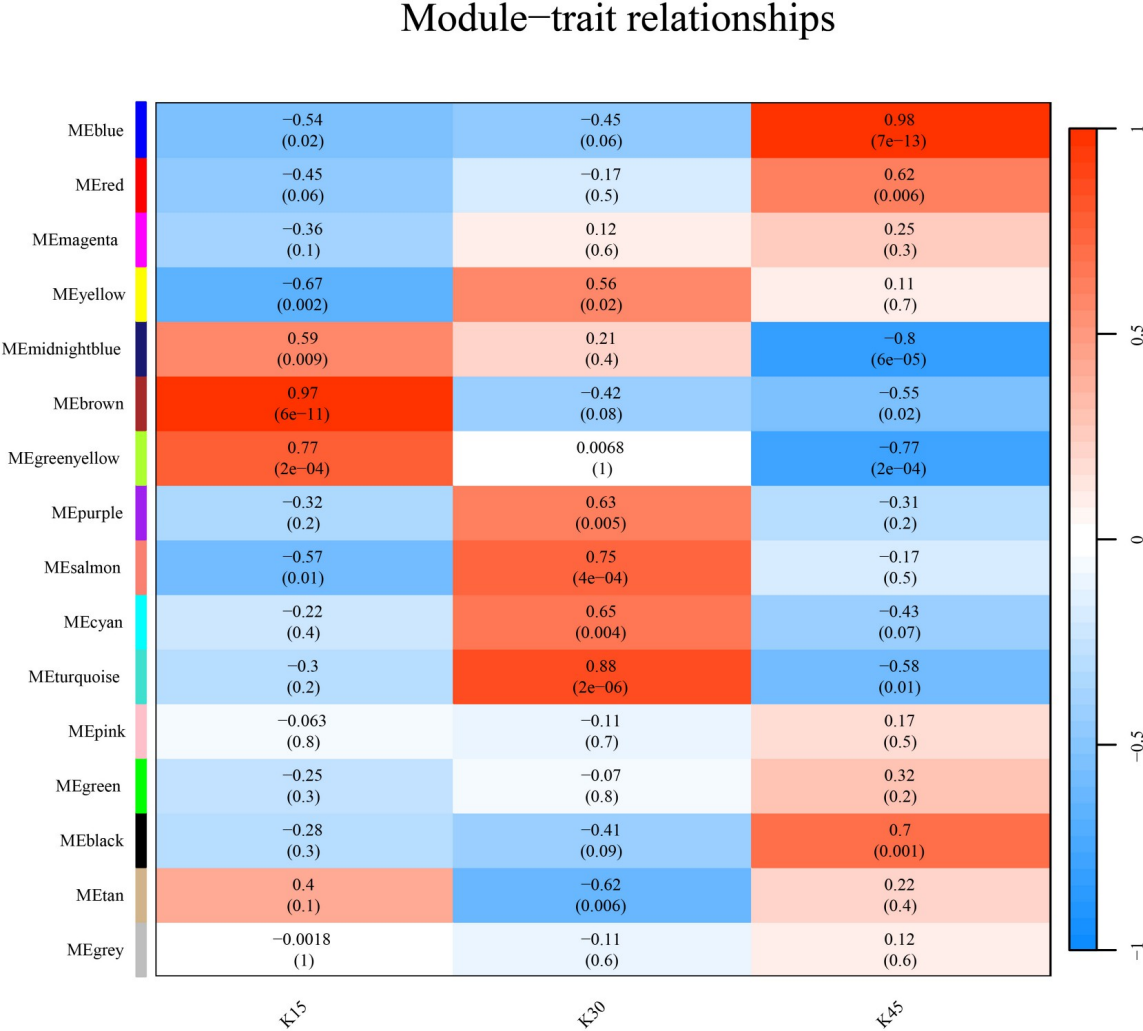
Different heatmap of module-trait associations.

**Fig 11:**
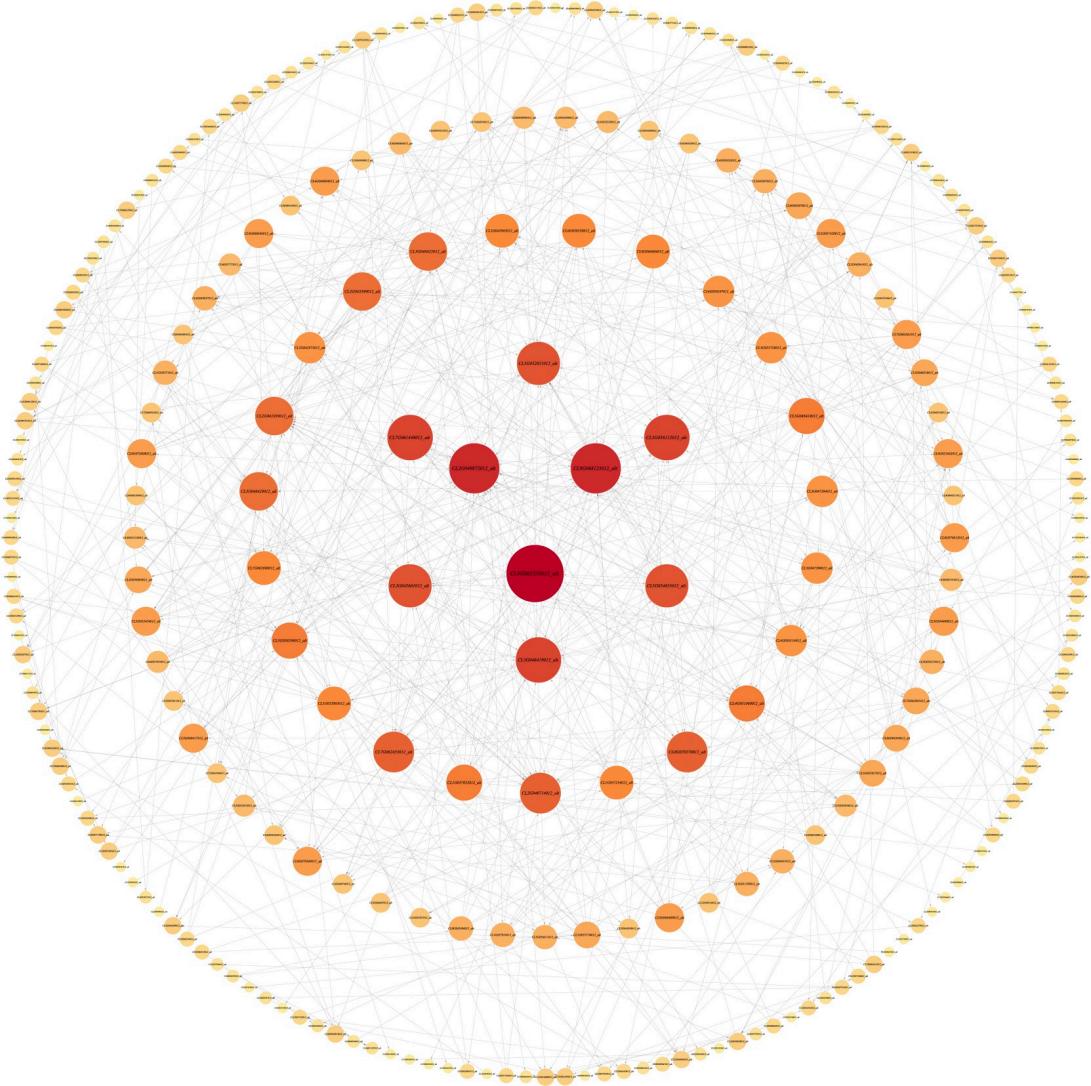
Gene coexpression network of the turquoise module.

Six of the 16 modules were highly specific to the sample, with two present at each defoliation period (Fig 10). To further resolve the biological functions of the modules specific to different defoliation stages, KEGG and GO functional enrichment analyses were performed on these six module genes in this study. The genes of the turquoise module were enriched to obtain a significant GO pathway (DNA binding transcription factor activity GO: 0003700, transcription regulator activity GO: 0140110, sequence-specific DNA binding GO: 0043565). Therefore, the present study focused on the turquoise module screened the genes with high connectivity and visualized the network using Cytoscape software (Fig 11). The genes in the network were the genes with high connectivity in the module. The Turquoise module identified sorbitol dehydrogenase (*CL9G068822012_alt*, *CL9G068820012_alt*, *CL9G068818012_alt*), abscisic acid 8’-hydroxylase (*CL8G064053012_alt*, *CL8G064054012_alt*), and asparagine synthetase (*CL8G065162012_alt*, *CL8G065151012_alt*) as the seven core genes.

### 3.8. RT‒qPCR validation of differentially expressed genes

**Fig 12:**
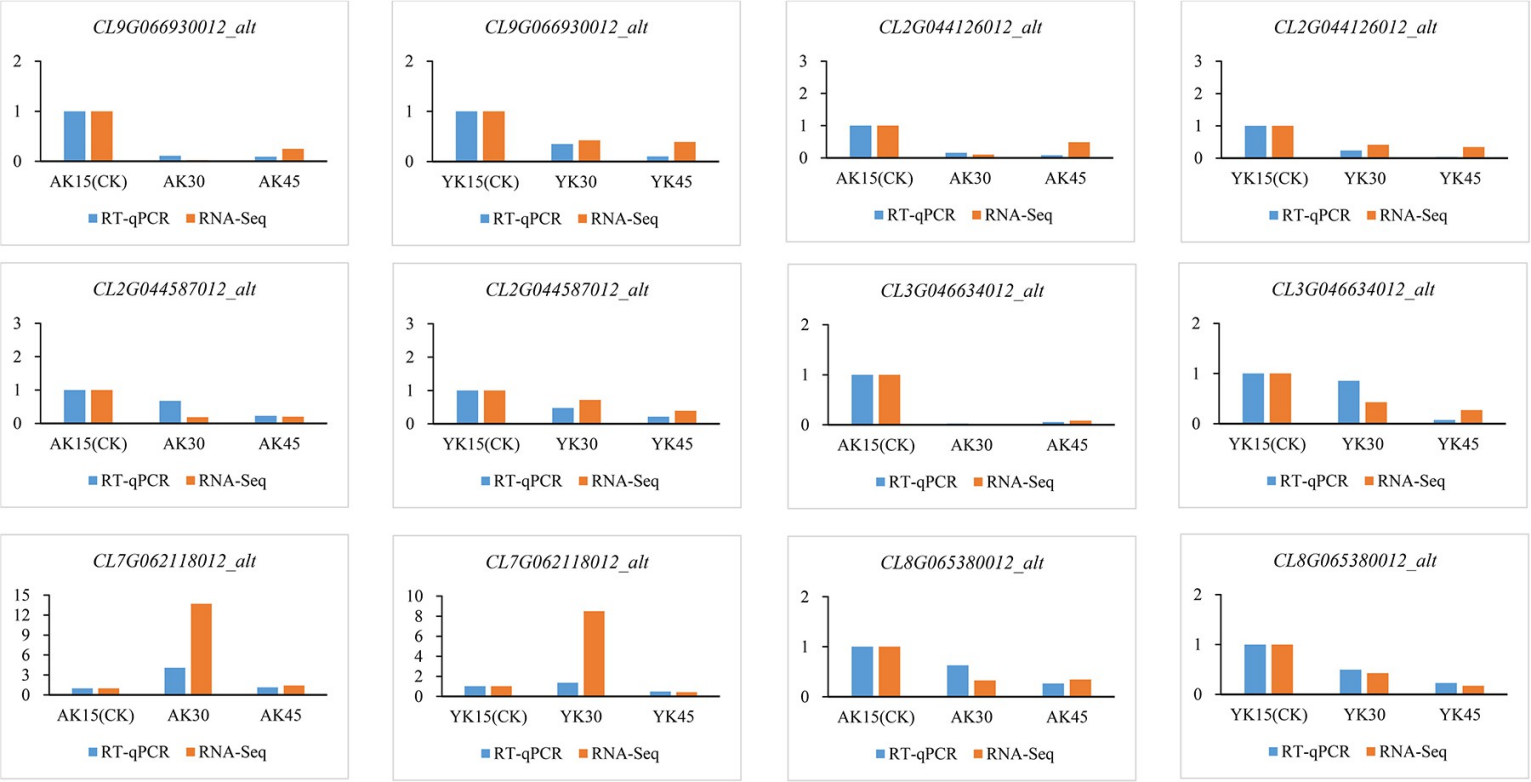
Validation of the expression levels of four differentially expressed genes.

In this study, we also used RT‒qPCR to validate six genes involved in plant hormone synthesis, signaling, and other processes related to plant organ abscission: hydrolases (*CL7G062118012_alt*), peroxidases (*CL2G044126012_alt*), weakly ethylene-insensitive proteins (*CL2G044587012_alt)*, Aux/IAA gene family (*CL3G046634012_alt*, *CL9G066930012_alt*) and protein phosphatase (*CL8G065380012_alt*). The results showed (as shown in Fig 12) that the changes in the fluorescence quantitative expression levels of the six differentially expressed genes converged with the changes in transcriptome gene abundance.

## 4. Discussion

Abscission is the shedding of entire nutrient and reproductive organs as a result of cell separation, a process that occurs in the site-specific abscission zone (AZ) of the plant body, and from an evolutionary point of view, abscission is a highly favorable process that leads to dispersal of fruits and seeds as well as the shedding of organs that are no longer useful. However, in agricultural production, shedding of large numbers of organs may become a major factor limiting crop yield [16–21].

In this study, we found by WGCNA that sorbitol dehydrogenase (*CL9G068822012_alt* and *CL9G068820012_alt*) did not show significant varietal differences in the prefoliation stage (k15). However, the expression level of ’Allen Eureka’ was significantly downregulated in the middefoliation stage (k30) compared to the prefoliation stage (k15), whereas the expression level did not change much in ’Yunning No. 1’. It suggests that sorbitol dehydrogenase genes associated with sugar metabolism may be involved in the regulation of lemon leaf defoliation. Sorbitol metabolism and transportation play a vital role in plant drought resistance and the improvement of fruit tree quality [22–26]. Many studies have shown that plants accumulate large amounts of fructose and sorbitol to maintain cellular osmotic pressure homeostasis when they experience drought, low temperature, high temperature, or some biotic stresses [27–29]. Sorbitol dehydrogenase (SDH) was first found in plants of the Rosaceae (apple) family. However, in recent years, SDH genes have also been identified in maize [30, 31], tomato [32], pear [33], loquat [34], peach [35], and Arabidopsis thaliana [36], suggesting that SDH may be widespread in the plant kingdom, and in the present study, the presence of sorbitol dehydrogenase was also found in lemon leaves. Under water stress, sorbitol metabolism is altered in peach trees, starting with a decrease in the activity of sorbitol dehydrogenase (the enzyme that degrades sorbitol) within the stem tip (reservoir organ), which leads to a decrease in the degradation of sorbitol in the stem tip, which in turn causes a decrease in the reservoir strength due to the accumulation of sorbitol within the stem tip, leading to a decrease in the amount of sorbitol transported from the ripening leaf, and ultimately the accumulation of sorbitol in the ripening leaf, which causes a decrease in the activity of sorbitol-6-phosphate dehydrogenase (the enzyme that synthesizes sorbitol) [37]. Jie et al. [38] utilized greenhouse potted 1-year-old apple trees (Malus domestica Borkh) and found that drought stress not only affected the plant’s uptake of nitrogen and phosphorus but also maintained a high concentration of sorbitol in the leaves for a long time. This suggests that sorbitol content is a physiological indicator closely related to drought tolerance in fruit trees. Kobashi et al. [22] suggested that the accelerated accumulation of sugars by ABA under water stress was initiated by activation of sorbitol metabolism. In pear leaves, NAD+-SDH activity was higher in young leaves and very low in mature leaves, but gene expression showed an opposite trend to the change in activity [39]. In this study, ’Allen Eureka’ lemon leaves away from the zone in the middefoliation stage (k30) sorbitol dehydrogenase expression is lower than the predefoliation stage (k15) and the postdefoliation stage (k45), may be at this time sorbitol dehydrogenase activity is higher on the contrary, and thus more sorbitol degradation, the decrease in the content of sorbitol, and can not continue to maintain the expansion of the pressure in the cell, resulting in cell dehydration, leaf abscission, and this time is the period of the largest amount of lemon leaf abscission.

In this study, the core genes ABA8’-hydroxylase (*CL8G064053012_alt*, *CL8G064054012_alt*) did not show significant differences among varieties at the three periods, but their expression levels were significantly upregulated at the middefoliation stage (k30) compared to the predefoliation stage (k15) and the postdefoliation stage (k45), as analyzed by WGCNA. It suggests that abscisic acid metabolism-related genes may play a vital role in lemon leaf abscission. Abscisic acid (ABA) is involved in the regulation of various physiological activities, such as seed dormancy, stomatal closure, organ abscission, and fruit ripening in plants [40–42]. Abscisic acid -8’-hydroxylase (ABAH) is a key enzyme catalyzing the catabolism of ABA, and its activity is closely related to the content of plant ABA and the high or low expression of its gene directly affects the homeostasis of ABA in the plant body [41]. When abscisic acid was first discovered, it was called abscisin II because it promotes organ shedding [43]. Later, it was shown by many studies that cotton abscission is highly correlated with abscisic acid [6]. Gomez-Cadenas found that water stress can cause the abscission of citrus leaves, which was tested and found to accumulate a large amount of abscisic acid in its roots [6]. Abscisic acid also increases cellulase activity and is involved in cell wall degradation [44]. The *CYP707A* gene encodes an ABA 8’-hydroxylase that negatively regulates endogenous ABA content and is important for plant growth and development under environmental stress conditions. Dehydration and rehydration induced a high expression of *AtCYP707A3*; up-regulation of *AtCYP707A3* expression decreased endogenous ABA content [45]. In the present study, the two lemon varieties in the middefoliation stage (k30) may be due to the large amount of abscisic acid produced after water stress acting on the petiole off-zone, and the dehydration of the leaf off-zone leading to leaf abscission, and at this time the dehydration of the off-zone petiole led to a significant up-regulation of the expression of ABA 8’-hydroxylase.

## 5. Conclusions

The results of this study showed that the metabolic pathways involved in lemon leaf abscission were mainly focused on plant hormone signal transduction, phenylpropanoid biosynthesis, amino sugar and nucleotide sugar metabolism, and flavonoid biosynthesis. And by WGCNA co-expression network analysis, found to encode Sorbitol dehydrogenase (*CL9G068822012_alt*, *CL9G068820012_alt*, *CL9G068818012_alt*), Abscisic acid 8’-hydroxylase (*CL8G064053012_alt*, *CL8G064054012_alt*), and asparagine synthetase (*CL8G065162012_alt*, *CL8G065151012_alt*), seven genes that may be involved in the leaf abscission response of lemon. above conclusions enrich the research related to lemon leaf abscission, provide reliable data for the screening of lemon defoliation candidate genes and the analysis of defoliation pathways, and provide a theoretical foundation and scientific basis for the selection and breeding of high-quality and highly resistant lemon varieties.

## Supporting information

S1 Table Transcriptome quality data sheet. (XLSX)

S2 Table Different varieties of differential genes. (XLSX)
